# Systematic Review: Measurement Methods and Concept of Resilience Among Seafarers

**DOI:** 10.1177/00469580231221288

**Published:** 2024-01-19

**Authors:** Wiebke Janssen, Hans-Joachim Jensen, Volker Harth, Marcus Oldenburg

**Affiliations:** 1 University Medical Center Hamburg-Eppendorf (UKE), Hamburg, Germany

**Keywords:** maritime, seafaring, resilience, hardiness, coping

## Abstract

Shipping is considered a demanding environment that can significantly impact seafarers’ well-being and mental health. This review aims to examine existing literature on the resilience of seafarers, with a focus on the measurement methods used. Furthermore, this study intends to gain a comprehensive understanding of the current state of research in the field of seafarers’ resilience, examining the variations in defining and conceptualizing resilience across different studies and contexts. The review identified 99 studies published between 2003 and 2023, with ten studies being included in the final analysis. These studies employed various measurement methods and provided definitions of resilience. Five questionnaires were identified, with the Dispositional Resilience Scale-15 (DRS-15) being the most commonly used. Two of the selected studies had a longitudinal follow-up, while eight were cross-sectional. Four studies related to tankers, and an additional four studies focused on naval vessels, while two studies did not specify the vessel type. The publications were distributed in the period between 2003 and 2013 (two papers) and between 2013 and 2023 (eight papers). The identified themes encompassed shipboard stressors (three papers), sleep problems (two papers), occupational groups or attitudes (two papers), experiences in war (two papers), and intervention measures (one paper), highlighting the multidimensional nature of resilience within the maritime field. This review suggests a research gap, as it reveals that the topic of resilience in seafaring has been sparsely represented. Despite an increasing interest in recent years, research remains limited, particularly in the civilian maritime sector. Therefore, this review highlights the importance of understanding and promoting resilience among seafarers. While the variety of questionnaires used was limited, achieving consensus and standardization in resilience measurement is essential for more comparable and consistent research findings. Recognizing resilience as a crucial resource can promote the development of targeted interventions and support systems, enhancing seafarers’ well-being and mental health.

**What do we already know about this topic?**
Regarding resilience in general, a substantial amount has been reported in the literature, highlighting the positive aspects of enhanced resilience among employees. However, the field of resilience among seafarers has received limited examination to date.
**How does your research contribute to the field?**
This systematic review highlights the lack of research and data on resilience in seafaring, emphasizing the need for further investigation.
**What are your research’s implications towards theory, practice, or policy?**
The aim of this review is to emphasize the necessity for a cohesive definition of resilience, enhance the importance of resilience in seafaring as a research subject, identify and evaluate measuring methods used and provide recommendations for appropriate measurement methods to facilitate further research initiatives.

## Introduction

Resilience is a construct for understanding how individuals cope with stress, adversity, and challenging situations.^1,2^ Resilience is a complex concept, and there is no universally accepted definition of resilience. The derivation of the term “resilience” comes from the Latin word “resilire,” which means to bounce back or rebound. The term “resilience” was originally used in the field of physics.^3^ The psychologist Jack Block was the first person to use the term “resilience” in his work with young children, where he examined ego-control and ego-resilience.^3^ One widely cited definition of resilience is provided by the American Psychological Association, which defines resilience as “the process of adapting well in the face of adversity, trauma, tragedy, or significant sources of stress—such as family and relationship problems, serious health problems, or workplace and financial stressors.”^4^ This definition emphasizes the importance of resilience as a process of adaptation, rather than a fixed trait.

The Leibniz Institute for Resilience Research (LIR) is a research center in Germany that focuses on the scientific study of resilience across the lifespan and describes resilience as an ability of individuals, and communities to cope with and adapt to adversity, to learn from challenges and setbacks, recover from stress and maintain or regain well-being and functioning.^5^ This definition highlights the importance of resilience as a multifaceted and dynamic process that involves not only coping with challenges and stressors but also learning and growth.

Hardiness is considered a protective factor that strengthens resilience, leading to higher life satisfaction and providing protection against mental illnesses.^6^ Hardiness is a stable personality trait encompassing elements such as commitment, control, and challenge, contributing to an individual’s ability to navigate difficult circumstances and maintain well-being. However, at the same time, resilience should be distinguished from hardiness and coping. Resilience encompasses the aspect of successful adaptation to stressors, particularly over time and across different situations, whereas hardiness is viewed as a stable personality trait contributing to resilience but representing a specific aspect of an individual’s overall resilience.^7^ The measurement of resilience is a complex and multifaceted process, and a wide range of methods to assess resilience have been developed and used in the literature. The selection of an appropriate measure for a given study depends on several factors, including the specific population being studied, the research question, and the psychometric properties of the measure.

In the context of seafaring, where individuals face working and living on a ship for extended periods of time, often under challenging and stressful conditions, involving prolonged separation from family and friends, long working hours, and exposure to extreme weather conditions, which can significantly impact the well-being and mental health of seafarers. These challenges can lead to heightened levels of stress, anxiety, and mental health issues among seafarers. In this context, resilience plays a crucial role in helping seafarers cope with and adapt to the various challenges they encounter during their work at sea. Resilience enables seafarers to bounce back from adversity, maintain their mental well-being, and effectively navigate the demanding maritime environment. Understanding and fostering resilience among seafarers are vital for promoting their mental health, safety, and overall well-being.^8,9^

To better comprehend resilience in this context, it is crucial to understand how resilience is defined and interpreted in this field and to have reliable and valid methods to measure resilience that are sensitive to the unique challenges in seafaring. In general, there are numerous methods and questionnaires available to assess resilience, but no tool has been established as gold standard or emerged as the predominant choice.^10^

Therefore, the present systematic review of the literature intends to identify the various measurement methods and evaluate the psychometric properties of different measures of resilience that have been used in the context of seafaring so far, as well as examining the variations in defining and conceptualizing resilience across different studies and contexts.

## Materials and Methods

A systematic literature search was conducted in February 2023 using the PubMed database to investigate resilience in seafaring in the period between 2003 and 2023. This timeframe was chosen due to the highest concentration of relevant studies during this period, making it the most meaningful for analysis. Pubmed was the only used database because of its relevance as a specialized biomedical literature database with comprehensive coverage and established credibility. Studies on resilience in seafarers were identified using the following search terms or MeSHTerms (Medical Subject Headings): (sailor*[Title/Abstract] OR seafarer*[Title/Abstract] OR seamen[Title/Abstract] OR seaman[Title/Abstract] OR naval[Title/Abstract]) AND (resilience OR coping OR self-efficacy OR coherence OR hardiness).

The following two inclusion criteria were established: exclusively scientific studies in German or English language as well as resilience in seafaring was defined and/or measured using specific methods. The term “seafarer” refers to individuals who stay on board ships, including naval personnel. The use of the SIGN (Scottish Intercollegiate Guidelines Network) criterion was considered to ensure a rigorous and standardized evaluation of the included studies, enhancing the reliability and credibility of this review. The SIGN criterion provides a well-established approach for assessing the quality of evidence in systematic reviews.

**Figure 1.**
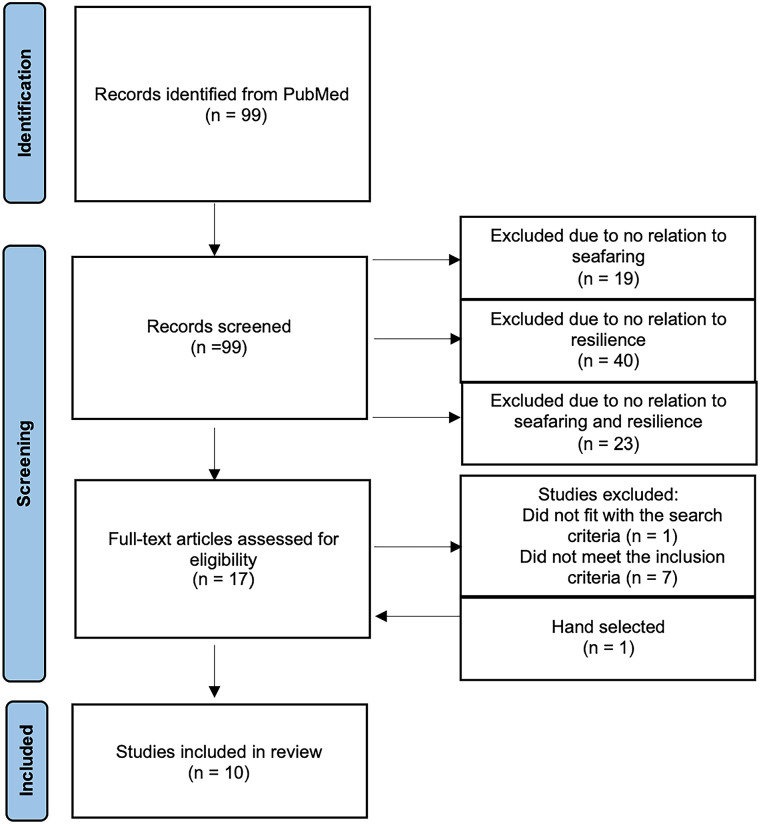
Flow chart for inclusion of studies.

During the observation period mentioned, the search string generated a total of 99 hits. Studies were selected based on the PRISMA (Preferred Reporting Items for Systematic Reviews and Meta-Analyses) statement. After screening the abstracts, 81 studies were excluded because they were not related to seafaring (n = 19), resilience (n = 39), or both (n = 23). In the subsequent full-text review one study was excluded because it focused solely on seafarers’ spouses rather than the seafarers themselves. Additionally, one study was hand-selected.^11^ A total of 7 further studies were excluded because resilience was neither defined nor measured in the full text.^12-18^ Finally, a total of 10 studies on resilience in seafaring were included in this review^8,11,19-26^ (Figure 1).

The studies were independently screened and evaluated by three different researchers. To compare various measurement methods in this review, Cronbach’s alpha as an internal consistency parameter was used in order to assess the suitability of different questionnaires for assessing seafarers’ resilience.

## Results

This section provides an overview of the selected studies, presenting general information about the age and gender distribution of the study populations as well as the types of vessels investigated. Additionally, the reasons for conducting the studies, the definitions of resilience used and the measurement methods as well as the resilience profiles are presented.

The present systematic review focused on ten studies measuring and/or defining resilience. Two of them were published in the period between 2003 and 2013,^20,22^ while eight papers were published between 2013 and 2023.^8,11,19,21,23-26^ Out of the ten studies, two had a longitudinal follow up,^19,25^ and the other eight were cross-sectional studies.^8,11,20-24,26^ Overall, the evidence level of these studies ranged from 2− to 2+ with eight studies meeting the SIGN criterion of 2−,^8,11,20,22-26^, two studies corresponding to the criterion of 2+.^19,21^ Populations varied from 18 to 3069 seafarers (Table 1).

**Table 1.** General Information of the Included Studies.

Author	Title	Study design	Sample size	Age/gender/marital status	Type of vessel	Reasons to carry out studies	Findings considering resilience	Recommendation level according to SIGN
McVeigh J. et al (2021)	Effects of an on-board psychosocial program on stress, resilience, and job satisfaction amongst a sample of merchant seafarers	Case-control study	122 seafarers (61 in each group; participants and matched control group)	Age 18-64; only men	Tanker	Impact of an intervention measure on seafarers’ resilience	The program and the number of weeks on board did not have an impact on participants’ levels of hardiness	2−
McVeigh J. et al (2019)	Identifying predictors of stress and job satisfaction in a sample of merchant seafarers using structural equation modeling	Longitudinal field study	512 seafarers responded at T0 (between January and July 2014), 276 seafarers responded at T1 (between November 2014 and March 2015)	Age 18-65+; 98.2% men	Tanker	Relationship between resilience/hardiness and shipboard stressors	Resilience significantly predicted job satisfaction and perceived stress	2−
Valdersnes K. B. et al (2017)	Does psychological capital moderate the relationship between worries about accidents and sleepiness?	Cross-sectional field study	397 maritime workers	age: <25 years: 12.2% 54< years: 9.5% 25-52 years: 78.3%; >99% men	Tanker	Relationship between resilience/hardiness and sleep problems	Worries about accidents did not interact significantly with resiliency	2−
Doyle N. et al (2016)	Resilience and well-being amongst seafarers: cross-sectional study of crew across 51 ships	Cross sectional field study	387 seafarers	Age 18-65; 98% men; seafaring experience 1-5 years	Tanker	Relationship between resilience/hardiness and shipboard stressors	Self-reported higher levels of resilience, longer seafaring experience and greater instrumental work support were significantly associated with lower levels of self-reported stress at sea total hardiness (all three components combined) predicted the dependent variable better than each component of hardiness (commitment, control, challenge) alone	2−
Nordmo M. et al (2017)	The effect of hardiness on symptoms of insomnia during a naval mission	Part of a longitudinal follow-up	281 marines (data was reduced to 164 because of missing data on one or more of the measurement intervals)	No information about the study population	Navy	Relationship between resilience/hardiness and sleep problems	Higher levels of hardiness were associated with lower levels of insomnia	2−
Bäccman C. et al (2016)	Improved resiliency and well-being among military personnel in a Swedish Naval Force after a counter-piracy operation off the coast of Somalia	Longitudinal field study	129 marines	Average age 31 years, ranging from 20 to 61 years; 120 men, 9 women	Navy	Relationship between resilience and war experiences	The members of ME01 (first Swedish Marine Force in the first European Union Naval Force) improved their resilience and decreased their stress reactions from T1 (before deployment) to T2 (after deployment). Members who reported several stressful events during ME01 reported a decrease in resiliency.	2−/2+
Mansfield A. J. et al (2011)	Suicidal or self-harming ideation in military personnel transitioning to civilian life	Cross sectional field study	3069 marines	Mean age: navy = 31.8 years; marine corps = 25.8 years; only men	Navy	Relationship between resilience and war experiences	A significant negative association between resilience and suicidal ideation, symptoms of depression, PTSD as well as substance abuse	2−
Eid J. et al (2004)	Stress and coping in a week-long disabled submarine exercise	Cross sectional field study	18 marines	age 23-43 years; only men	Navy	Relationship between resilience/hardiness and shipboard stressors	Personality hardiness emerged as a resilience factor in relation to the experience of emotional stress personality hardiness was negatively associated with emotional stress and lower quality of life	2−
Makarowski R. et al (2020)	The human factor in maritime transport: personality and aggression levels of master mariners and navigation students	Cross sectional field study	76 captains; 108 navigation students	Mean age of navigation students = 21.4 years mean age of master mariners = 55.1 years; only men	Not further described	Resilience as a feature of occupational groups or attitudes	Captains mainly exhibit the resilient personality type; navigation students exhibit different personality types	2+
Bergheim K. et al (2015)	The relationship between psychological capital, job satisfaction, and safety perceptions in the maritime industry	Cross-sectional field study	S1: 486 maritime workers S2: 594 maritime workers	S1: mean age = 40.8 years; only men S2: mean age = 40.0 years; 99% men	Not further described	Resilience as a feature of occupational groups or attitudes	S1 (Study 1): officers and non-officers perceived the safety climate as similar when their PsyCap is low officers with high level of PsyCap have a more positive perception of the safety climate than non-officers with high levels of PsyCap S2 (Study 2): a positive association was established between safety perceptions and job satisfaction, as well as between PsyCap and job satisfaction	2−

### Age and Gender

Among the ten included studies, detailed information about the study population was provided in nine studies, while one study lacked comprehensive details.^14^

Regarding gender representation, eight studies provided information on both age and gender, while one study^22^ only indicated the gender.^8,11,19-21,23,24,26^ The age range of the study populations spanned from 18 years^8,23,24^ to >65 years.^24^ Additionally, four studies reported mean ages ranging from 21.4 to 55.1 years.^11,19,21,22^ Four out of the ten studies exclusively included male study populations,^20-23^ while five studies had a small proportion of women,^8,11,19,24,26^ with the highest percentage of women (7%) observed in the study by Bäccman et al (Table 1).^19^

### Type of Vessel

When examining the vessels used in the studies, a clear grouping was evident, as four out of the ten studies investigated crews from tankers,^8,23,24,26^ four investigated naval vessels.^19,20,22,25^ In two out of the ten papers, it was not possible to determine the specific type of ships.^11,21^

### Reasons to Carry Out Studies

The different reasons for conducting the studies could be categorized into five groups. Three studies^8,20,24^ focused on investigating the association between resilience/hardiness and shipboard stressors. Two studies^25,26^ examined the relationship between resilience/hardiness and sleep problems. Two studies^11,21^ investigated resilience as a characteristic of occupational groups or attitudes. Two studies^19,22^ explored the connection between resilience and experiences in war. Lastly, one study^23^ investigated the impact of intervention measures on seafarers’ resilience.

### Definitions of Resilience

Among the ten studies included, nine provided definitions or descriptions related to resilience. These definitions and descriptions can be categorized into three distinct groups as presented in Table 2. Group 1 encompasses definitions that conceptualize resilience as an individual’s characteristic or positive psychological capacity to rebound from adversity and the ability to adapt and overcome various challenges and demands. This particular understanding of resilience was identified in six different studies.^8,19,21-23,26^ Group 2 highlights the significance of hardiness as a component of resilience and includes definitions from four different studies.^8,20,23,25^ The third group accentuates the connection between resilience and related concepts such as Antonovsky’s concept of Sense of Coherence^19^ and the Psychological Capital.^11^ Resilience was linked to these specific concepts in two different studies.^11,19^ Among the nine studies that incorporated definitions of resilience, a distinct pattern emerged in their usage. Specifically, three papers included aspects of descriptions of resilience from two different groups,^8,19,23^ indicating a comprehensive approach to defining resilience. The other six studies relied on only one single definition to shape their conceptualization of resilience.^11,20-22,25,26^

**Table 2.** Definitions of Resilience in Groups.

Group 1: Resilience as a psychological capacity/characteristic and adaptability	Group 2: Hardiness as a facet of resilience	Group 3: Resilience and related concepts
- Resilience is defined as the ability to “bounce back” from adversity.^8^ - Resilience is defined as the ability to cope despite repetitive and long-lasting or chronic demands.^8^ - Resilience is described as an individual characteristic and a process.^19^ - Resilience is defined as the positive psychological capacity to rebound from various challenges and changes.^23^ - Resilience is described as adaptability and the ability to bounce back from harm rather than being immune to it.^23^ - The concept of psychological resilience encompasses qualities that allow an individual to cope with stress and persevere during adversity.^22^ - Resilient individuals as those with strong impulse control, assertiveness, openness to different perspectives, and the ability to manage emotions. They maintain focus on optimal task performance even in challenging situations, without exhibiting aggression in interpersonal or task-oriented scenarios.^21^ - People with a higher score on resiliency tend to adapt better.^26^	- Hardiness is considered a personality disposition and a resiliency factor.^25^ - Hardiness is characterized as a facet of resilience.^8^ - Personality hardiness is seen as a possible trajectory to resilience, characterized by attitudes and skills that support resilience and thriving under stress.^23^ - Personality hardiness is assumed to emerge as a factor of resilience.^20^	- Antonovsky’s concept of a sense of coherence is mentioned as a recurring aspect of resilience.^19^ - Posttraumatic growth (PTG) is described as another concept of resilience.^19^ - Resiliency as a factor of PsyCap: Resilience empowers individuals to flourish through positive adaptations to change, enabling workers to feel comfortable outside their usual comfort zone and confidently confront personal assumptions and external obstacles.^11^

### Measurement Methods

All of the ten included studies employed specific measurement methods to assess the levels of resilience among seafarers. These measurement methods encompassed the utilization of five distinct questionnaires, namely the Dispositional Resilience Scale (DRS-15), Antonovsky’s 13-item Scale of Sense of Coherence (SOC-13), assessments of the Big Five Personality Traits, the Psychological Capital Questionnaire (PCQ), and the Connor-Davidson Resilience Scale (CD-RISC) (Table 3).

**Table 3.** Definitions and Scales of the Included Studies.

Author	Used definition of resilience	How was data about resilience collected?	numerical values of the scale	Reason to use method/questionnaire internal consistency
McVeigh J. et al (2021)	Resilience is defined by Luthans as the “positive psychological capacity to rebound, to ‘bounce back’ from adversity, uncertainty, conflict, failure or even positive change, progress and increased responsibility.” Resilience is also defined as an adaptability, as a progress of bouncing back from harm instead of immunity from harm. Personality hardiness is seen as a possible trajectory to resilience, characterized by attitudes and skills that support resilience and thriving under stress.	Dispositional Resilience Scale-15 (DRS-15)	/	They decided to use the DRS-15 because of its established validity, acceptable reliability, as well as brevity DRS-15 uses positive and negatively keyed items the scale measures hardiness; hardiness and resilience were considered closely associated Cronbach’s alpha = .70 (T0) and .73 (T1)
McVeigh J. et al (2019)	/	Dispositional Resilience Scale-15 (DRS-15)	/	They decided to use the DRS-15 based on its established validity, acceptable internal consistency, acceptable test-test reliability and its brevity usage of the total resilience score instead of the subscales due to below acceptable values of internal consistency of the subscales in previous studies Cronbach’s alpha = .70 (T0 - before pilot resiliency program) 0.73 (T1 - after pilot resiliency program) Cronbach’s alpha (internal consistency) =.83 (Bartone)
Valdersnes K. B. et al (2017)	People with a higher score on resiliency tend to adapt better.	12-item Psychological Capital Questionnaire (PCQ-12) to measure PsyCap, including 3 items for resiliency	Mean PsyCap = 4.72 (S1) mean PsyCap = 4.82 (S2)	People with a higher score on resiliency tend to adapt better when they experience changes or setbacks the shorter version of the scale was used to reduce the response burden of the seafarers Cronbach’s alpha for the PCQ-12 = 0.9
Doyle N. et al (2015)	Resilience is defined as the ability to “bounce back” from adversity (Luthans et al. 2006). “the ability to cope despite repetitive and long-lasting or chronic demands.” hardiness as a facet of resilience (stable personality trait)	Dispositional Resilience Scale (DRS-15)	Mean DRS score of 30.4	Cronbach’s alpha was 0.72 for total hardiness in present sample only total hardiness was used (combination of the subscales commitment, control, challenge) because the internal consistency was 0.65/0.57/0.57
Nordmo M. et al (2017)	Hardiness is considered a personality disposition and a resiliency factor.	15-item Dispositional Resilience Scale (DRS-15-R); 2 groups: scores below 49 (n = 79) as lower hardiness group and scores over 49 (n = 88) as higher hardiness group	Mean DRS-score of 48.2 mean DRS-score in the high hardiness group: 51.8 mean DRS-score in the low hardiness group: 44.6	DRS-15-R was recommended by Funk as the best measuring device for hardiness measuring hardiness is suggested as a valuable tool in personnel selection because of its sensitivity to resilience factors Cronbach’s alpha = .73 (Funk)
Bäccman C. et al (2016)	Resilience is described as an individual characteristic and a process. Antonovsky’s concept of a sense of coherence is mentioned as a recurring aspect of resilience. Posttraumatic Growth (PTG) as another concept of resilience	Coherence was measured using Antonovsky’s 13-items scale of sense of coherence (SOC)	Mean SOC-13 at T1 was 69.0 mean SOC-13 at T2 was 73.7	Cronbach’s alpha for SOC-13 = 0.78 (T1) and 0.81 (T2)
Mansfield A. J. et al (2011)	The concept of psychological resilience encompasses those qualities that allow an individual to cope with stressful situations and persevere during times of adversity.	25-item Connor-Davidson Resilience Scale (CD-RISC) the scale builds on the work of previous researchers on hardiness, action orientation, self-efficacy, confidence, adaptability, patience, and endurance in the face of adversity	Resilience = 76.2 (16.0) (navy) resilience = 72.8 (16.6) (marine corps)	The CD-RISC demonstrates good internal consistency and test-test reliability in general population and clinical samples good correlation with measures of stress and hardiness reflects different levels of resilience in populations that are thought to be differentiated by their degree of resilience Cronbach’s alpha = .89 (not self measured)
Eid J. et al (2004)	Personality hardiness is assumed to emerge as a factor of resilience.	Norwegian translation of the Dispositional Resiliency scale (DRS)	Mean DRS score of 31.3	The DRS-15 has shown good reliability and validity across a wide range of samples Cronbach’s alpha in the present example was 0.61
Makarowski R. et al (2020)	Resilient individuals as those with strong impulse control, assertiveness, openness to different perspectives, and the ability to manage emotions. They maintain focus on optimal task performance even in challenging situations, without exhibiting aggression in interpersonal or task-oriented scenarios.	The Big Five personality traits were measured using the NEO-FFI (neuroticism, extraversion, openness, conscientiousness, agreeableness) the resilient personality type was characterized by low neuroticism, high extraversion and conscientiousness	/	Cronbach’s alpha: neuroticism = .80, extraversion = .77, openness = .68, agreeableness = .68, and conscientiousness = .82
Bergheim K. et al (2014)	Resiliency as a factor of PsyCap: Resilience empowers individuals to flourish through positive adaptations to change, enabling workers to feel comfortable outside their usual comfort zone and confidently confront personal assumptions and external obstacles	24-item PCQ with six items for each subscale of efficacy, hope optimism and resiliency	Mean PsyCap = 5.06	Cronbach’s alpha for the overall PsyCap scale = .78

The Dispositional Resilience Scale (DRS-15) was the most commonly used questionnaire and appeared in five different studies.^8,20,23-25^ It consists of a four-point scale and includes positively and negatively keyed items. The internal consistency of the scale varied across the detected studies with Cronbach’s alpha ranging from 0.61^20^ to 0.83.^24^ The total hardiness score of this questionnaire (combination of the three subscales for commitment, control, challenge) was often used instead of single subscales due to described lower internal consistency values for the subscales in previous studies.^8,23,24^

Antonovsky’s 13-item Scale of Sense of Coherence (SOC-13) was only used by Bäccman et al comprising a set of 13 items.^19^ The SOC-13 was employed as a measurement tool to assess the sense of coherence construct, which can serve as an indicator of resilience.^19^ The Cronbach’s alpha ranged from 0.78 to 0.81 across different points of time (before and after deployment).^19^ Makarowski et al^21^ considered the seafarers’ Big Five Personality traits using the NEO-FFI, including neuroticism, extraversion, openness, agreeableness, and conscientiousness to examine resilience in seafaring populations. Resilient personality types were characterized by low neuroticism, high extraversion, and conscientiousness. The Cronbach’s alpha values for all of the five traits ranged from 0.68 to 0.82.^21^ Two versions of the Psychological Capital Questionnaire (PCQ), including the shortened form PCQ-12,^26^ and the original long version PCQ-24,^11^ were employed in the studies. The PCQ-12 measured psychological capital, including three items related to resiliency, while the PCQ-24 included six resiliency-related items. In order to alleviate the response burden placed on seafarers, the abbreviated version of the scale was utilized.^26^ The internal consistency of the PCQ-12 was reported to be 0.9,^26^ and the overall scale of the PCQ-24 had a Cronbach’s alpha of 0.78.^11^ The Connor-Davidson Resilience Scale (CD-RISC), a 25-item scale, was used by Mansfield et al to measure resilience in seafaring populations.^22^ The decision to utilize the CD-RISC was made on the basis of its known robust internal consistency and test-retest reliability, as supported by previous studies conducted on both general population and patients.^27^ Specifically, the CD-RISC exhibited a high level of internal consistency, with a reported Cronbach’s alpha coefficient of 0.89.^22^

### Findings of Resilience Among Seafarers

Out of the five studies using the DRS-15, three studies documented concrete hardiness scores for the study population.^8,20,25^ Eid et al reported a mean DRS-score of 31.3 for the 18 marines.^20^ Nordmo et al reported a mean DRS-score of 48.2 for the entire study population and divided the marines into a high hardiness group with a mean DRS-score of 51.8 and a low hardiness group with a mean DRS-score of 44.6.^25^ The lowest DRS-score with a value of 30.4 was documented in the study population of Doyle et al .^8^ McVeigh et al did not report specific values in both of their studies.^23,24^

Using Antonovsky’s 13-items scale of sense of coherence (SOC), Bäccman et al recorded SOC-13 scores at two different points in time. At T1 (before deployment) the SOC-13 was 69.0 and at T2 (after deployment) was 73.7.^19^ According to the 25-item Connor-Davidson Resilience Scale (CD-RISC), Mansfield et al measured a resilience score of 76.2 for the navy group and a score of 72.8 for the marine corps group.^22^ The Psychological Capital Questionnaire (PCQ) was used in two different versions. With the PCQ-12, mean PsyCap scores of 4.72 and 4.82 were measured.^26^ Bergheim et al measured a PsyCap score of 5.06 using the PCQ-24.^11^

### Excluded Studies

Seven maritime studies were excluded at the end of the present literature search because—although resilience was mentioned in the text—it was neither defined nor assessed with a valid instrument.^12-18^ Nevertheless, some information can be extracted from these papers. Of the seven studies, two studies also examined populations from the navy.^12,18^ Two papers investigated study populations from commercial ships,^16,17^ one paper included any type of vessel,^13^ and in one paper the type of vessel was not specified.^14^ One paper is not a field study like the other six but rather a review, so no vessel can be indicated here.^15^ Three out of the seven studies mentioned social support as a factor positively influencing resilience.^12,16,17^ Furthermore, four out of the seven studies primarily focused on the impact of COVID-19 on mental health or work stress.^13,14,16,17^

## Discussion

The present systematic review aims to assess the measurement methods and relevance of resilience among seafarers through the analysis of the ten selected studies. The findings shed light on various aspects related to the definitions of resilience, measurement methods, profiles of resilience, characteristics of the study populations and reasons for conducting the studies. The studies addressed mental health, stress management and wellbeing in relation to resilience in seafarers.

Two of the ten included papers were published between 2003 and 2013,^20,22^ while eight papers were published between 2013 and 2023.^8,11,19,21,23-26^ This suggests an increasing interest in recent years in resilience within the maritime context, indicating its growing recognition as a relevant and significant topic in the field of maritime medicine and occupational health.

When examining the study populations of the papers, it is noticeable that the focus has primarily been on the military and the crews of tankers. The civilian maritime sector, apart from tankers, including most importantly container shipping as well as cargo shipping and passenger shipping has been completely overlooked in this regard. It is essential to promote research in this domain as well, given that the stressors within various sectors of the shipping industry can vary greatly. This divergence can lead to different issues, influencing mental health and the development of resilience.

### Definitions of Resilience

Regarding the definitions of resilience, the included studies demonstrated a diversity of conceptualizations. Three distinct groups of definitions emerged from the analysis. As shown in Table 2, these findings underscore the multidimensional nature of resilience and the varying perspectives within the maritime field.^11,19^ The definition of resilience provided by the Leibniz-Institute for Resilience Research (LIR), based on their renowned and extensive research, is considered the most suitable for the present review.^5,28^ The LIR’s definition encompasses the key aspects of resilience: optimism, self-efficacy, and social support. Resilience is described as an internal and external learning process that is changeable and represents a growth process. In summary, according to the LIR’s definition, resilience refers to the ability to maintain or rapidly recover good mental health despite experiencing stress or adversity. It involves employing successful methods and strategies to overcome crises and difficult life situations. Resilience factors, such as an optimistic mindset, high expectation of self-efficacy, and a supportive social environment, contribute to activating resilience mechanisms.^5^ The goal of resilience research is to identify these factors, mechanisms, and adaptation processes in order to develop interventions that prevent stress-related mental illnesses, particularly for individuals with low resilience factors or additional risk factors.^5^

The definitions of Group 1 align most closely with the definition of the LIR. The definitions of Group 2, which mainly focus on hardiness as a factor of resilience, capture only one aspect of resilience, but do not encompass it as a whole, as hardiness itself should be distinguished from resilience.^7^ The third group does not consider resilience as a separate concept but rather as an element of a broader concept, which makes it appear less central compared to the other definitions. Additionally, other concepts such as Antonovsky’s concept of a sense of coherence and Posttraumatic growth are presented as part of resilience in this group, which only represents certain aspects of resilience but not the concept as a whole. The most comprehensive perspective on resilience in maritime medicine is provided by the 3 papers that include definitions aligning with both Group 1, which is closest to the definition of the LIR, and additionally another group.^8,19,23^

### Measurement Methods

The included studies employed various measurement methods to assess resilience among seafarers. The most commonly used questionnaire was the Dispositional Resilience Scale (DRS-15), indicating its relevance in capturing resilience/hardiness levels in a maritime setting. However, the Cronbach’s alpha for the DRS-15 indicated variability in its internal consistency across different studies, with the lower value raising some doubts about its reliability. This suggests that researchers should exercise caution when interpreting hardiness scores using the DRS-15 and consider potential limitations in its consistency as a measurement tool.

Other measurement methods, such as the SOC-13, the Big Five personality traits (NEO-FFI),^21^ the PCQ-12, and the PCQ-24, have demonstrated varying levels of internal consistency in different studies.^11,26^ The SOC-13^19^ showed acceptable internal consistency, while the NEO-FFI had inconsistent reliability with scores ranging from unacceptable to good. The PCQ-12 exhibited strong internal consistency, but this conclusion is based on a single study. Mansfield et al did not report an internal consistency for the CD-RISC in their present sample but relied on a good Cronbach’s alpha of .89 for the scale, reported in a different study.^22,27^

The DRS-15 was recommended as the best measuring device of hardiness by Funk^29^ but had the weakest internal consistency among the assessed instruments. This questionnaire measures psychological hardiness and distinguishes between individuals who remain healthy under stress and those who develop stress-related problems based on commitment to life, control in life, and willingness to overcome challenges. The questionnaire consists of 15 items rated on a 4-point Likert scale from 0 (not at all true) to 3 (completely true),^30,31^ total score ranging from 0 to 60, with higher score indicating greater resilience.^32^

Antonovsky’s Sense of Coherence scale uses 13 items rated on a 7-point Likert scale to assess how people view life and identify how they use their resistance resources to maintain and develop their health. The total score ranges from 13 to 91 points, with a higher score indicating greater resilience. The CD-RISC questionnaire contains a 25-item scale, with each item rated on a 5-point scale. The total possible score ranges from 0 to 100 points. The questionnaire measures several components of resilience, including the ability to adapt to change, deal with challenges, cope with stress, stay focused and think clearly, not get discouraged in the face of failure, and handle unpleasant feelings such as anger, pain, or sadness.^33^

The PCQ (Psychological Capital Questionnaire) assesses the four dimensions of self-efficacy, hope, optimism, and resilience.^34^ The Big Five personality traits are measured using the NEO-FFI, which assesses the five personality traits: neuroticism, extraversion, agreeableness, openness, and conscientiousness. It consists of 60 items. In total, determining the most suitable questionnaire to measure resilience depends on various factors, including the specific research or intervention goals, the population being assessed and the psychometric properties of the questionnaire. The CD-RISC and the DRS-15 are some widely used and well-established questionnaires to measure resilience/hardiness. As the literature search in this present review shows, the DRS-15 is the most used questionnaire to measure resilience/hardiness in the maritime context and therefore comparison collectives are available.^8,20,23-25^ It is important to note that no single questionnaire can be considered universally superior for measuring resilience. Researchers and practitioners should carefully consider the specific aspects of resilience they aim to assess, the availability of comparison collectives and the psychometric properties of the questionnaire. The CD-RISC assesses multiple components of resilience, including the ability to adapt to change, cope with stress, stay focused, and think clearly. It aligns with the LIR’s definition by measuring various aspects of resilience. Similarly, the DRS-15 focuses on psychological hardiness, which includes commitment to life, control in life, and willingness to overcome challenges. These dimensions are related to the LIR’s definition of resilience and the factors that contribute to resilience but only cover one part of resilience. Therefore, the CD-RISC seems to be the best questionnaire to obtain a comprehensive impression of resilience among seafarers in the study cohort. However, if the interest is in comparing a study cohort of seafarers with other cohorts, the DRS-15 is most suitable, as it has been most frequently used in this context and has several comparison cohorts available. One assessment method that was not used in any of the studies included in this systematic review but is recommended by Kunzler et al is the Brief Resilience Scale.^35^ According to Kunzler et al considering stressor exposure is essential for properly assessing resilience. The Brief Resilience Scale is a measurement method that captures the ability to recover from stress, which is a unique feature.^35^ Therefore, it may be of particular interest to use the Brief Resilience Scale in future maritime health research. Additionally, Kunzler et al call for more longitudinal studies due to the dynamic nature of resilience, for which the Resilience Score (R-Score) would be suitable for data collection.^35^ The R-Score would involve at least two measurement points and consider the (job-related) stressor exposure that is particularly highly important in maritime context.

### Findings of Resilience Among Seafarers

Five studies used the 15-item Dispositional Resilience Scale (DRS-15) to measure hardiness. Eid et al found a mean DRS-score of 31.3 for 18 marines,^20^ while Nordmo et al reported a higher mean score of 48.2 for the overall study population, with high (mean score of 51.8) and low (mean score of 44.6) hardiness groups.^25^ Doyle et al reported the lowest score of 30.4.^8^ To compare, a mean DRS of 27.9 was measured in a group of nurses,^36^ which is lower than the DRS values recorded for seafarers in this systematic review, corresponding to higher resilience/hardiness of maritime personnel. One possible reason for the higher resilience/hardiness among seafarers could be attributed to cultural and/or situational social desirability bias in their response patterns, for example, notions of masculinity or societal expectations. In this case, the research findings may be called into question.^37^ Another possibility is that seafarers may possess higher resilience/hardiness due to the presence of resilience factors such as religiosity and religious practices, as well as positive affiliation with their own ethnic group.^38^ Bäccman et al indicated a slight increase in coherence following deployment, using the SOC-13.^19^ The latter could indicate a potential adaptation and adjustment to the situation, as well as possibly enhanced resilience through coping with stressful situations over the course of maritime time. In comparison to general population, a SOC-13 scale mean value of 67.3 was found for men aged 18 to 40 years in a study, and 66.8 for men aged 41 to 60 years.^39^ These reference values are similar to the measured values for seafarers. The reference value for the CD-RISC questionnaire is 80.7 for the US general population, therefore the sailors in the study of Mansfield et al^22^ fall slightly lower than the US general population.^40^ In summary, it can be concluded that the results regarding resilience among seafarers do not represent a clear picture but are rather reflective of the heterogenous nature of the study samples. Considering the included studies there is a lack of detailed information on cultural differences and other factors that could influence resilience. Each specific environment within the seafaring context may exhibit its own consistent results, emphasizing the importance of considering and accounting for these unique factors in future research.

Particularly, individuals in the Asian cultural context, according to the concept proposed by Frey and Jonas, tend to mentally engage with seemingly chaotic, unclear, and uncontrollable situations only when action is absolutely necessary.^41^ Unfortunately, the studies did not provide any insights into internal and external factors, for example, contact to family or internet availability. Only 3 studies that were finally excluded due to neither providing a definition of resilience nor using a questionnaire for measurement named social support as a known positive factor influencing resilience, but no further data on resilience were collected.^12,16,17^ To address this gap, researchers should consider incorporating additional measures or data collection methods in their studies to explore these factors more comprehensively. For example, qualitative research methods, such as interviews or focus groups, could be employed to gather in-depth insights into seafarers’ experiences and perceptions related to family contact, internet availability, community support, coping strategies, and self-control. Furthermore, longitudinal study designs that capture changes in their resilience profiles and experiences in response to various internal and external factors would provide valuable information. By incorporating a more comprehensive approach to understanding resilience among seafarers, future research can contribute significantly to the development of targeted interventions and support systems that promote resilience and well-being in this demanding and unique occupational setting.

### Limitations

It must be noted that this systematic review has certain limitations. Firstly, the literature search was conducted based on only one database, which may result in limitations in the search results and potential omission of some available papers. Secondly, a significant portion of the included studies focused on the navy as study population, thus predominantly covering this specific niche area. Furthermore, the studies did not provide any insight into internal and external factors therefore, it is not possible to state clearly how different profiles of resilience evolved. Additionally, this systematic review encompasses only a relatively small number of studies, which is attributed to the limited literature available on this topic. As a further limitation, due to the small number of contributions and data on the questionnaires on resilience in maritime medicine, no general statements can be derived on the resilience of seafarers.

## Conclusions

This systematic review revealed a scarcity of research on resilience in seafaring, indicating a lack of attention given to this topic in the literature. The findings demonstrated that existing research primarily focuses on tankers and naval vessels, while other areas of seafaring remain understudied. The included studies utilized various definitions and measurement methods of resilience, demonstrating the complexity and multifaceted nature of the construct. In total, no universally approved measurement tool has emerged. Choosing the right resilience questionnaire depends on research goals, the target population, and properties. CD-RISC and DRS-15 are most often used in seafaring. In the maritime context, DRS-15 allows comparisons, however, it focuses on psychological hardiness as only one part of resilience. CD-RISC offers a comprehensive view and is best for overall impression. The results regarding resilience among seafarers are inconsistent, and the diversity of findings suggests the existence of distinct resilience profiles within different contexts of seafaring, highlighting the importance of considering the unique challenges and characteristics of each maritime environment. This finding underscores the need for additional studies to better understand resilience in the context of seafaring and to develop reliable and valid measures that capture the unique challenges faced by seafarers. By enhancing the understanding of resilience in seafaring populations, future research can contribute to the well-being, mental health and safety of seafarers and promote interventions and policies that support their resilience and adaptability in challenging maritime environments.
